# Dynamics of SERPINA3 in response to anthracycline treatment and cardiovascular dysfunction

**DOI:** 10.1186/s40959-025-00324-7

**Published:** 2025-03-14

**Authors:** Hanne M. Boen, Lobke L. Pype, Konstantinos Papadimitriou, Sevilay Altintas, Laure-Anne Teuwen, Sébastien Anguille, Kirsten Saevels, Anke Verlinden, Leen Delrue, Ward A. Heggermont, Matthias Bosman, Pieter-Jan Guns, Hein Heidbuchel, Caroline M. Van De Heyning, Emeline M. Van Craenenbroeck, Constantijn Franssen

**Affiliations:** 1 Research Group Cardiovascular Diseases, GENCOR, University of Antwerp, Antwerp, Belgium; 2 Department of Cardiology, Antwerp University Hospital, Antwerp, Belgium; 3 Department of Oncology, Antwerp University Hospital, Antwerp, Belgium; 4 Department of Haematology, Antwerp University Hospital, Antwerp, Belgium; 5 Department of Cardiology, Olv Hospital Aalst, Aalst, Belgium; 6 Research Group Physiopharmacology, GENCOR, University of Antwerp, Antwerp, Belgium

**Keywords:** Cancer therapy-related cardiac dysfunction (CTRCD), SERPINA3, Cardiotoxicity, Biomarker, Anthracycline, Breast cancer

## Abstract

**Background:**

SERPINA3 recently emerged as potential prognostic biomarker in heart failure. In a population of cancer survivors with cancer therapy-related cardiac dysfunction (CTRCD) circulating SERPINA3 was elevated compared to age-matched controls. We aimed to assess the longitudinal dynamics of circulating SERPINA3 levels in patients with cancer treated with anthracycline chemotherapy (AnC) and its relation to CTRCD.

**Methods:**

In this single centre cohort study, 55 patients with cancer scheduled for AnC were prospectively enrolled. Cardiac evaluation (echocardiography, high-sensitive cardiac troponin I and NT-proBNP) was performed and SERPINA3 levels in plasma were assessed at 4 timepoints: before chemotherapy, directly after the end of chemotherapy, three months and twelve months after the end of chemotherapy.

**Results:**

Forty-two out of 55 patients (76.4%) developed CTRCD within 1 year after end of treatment. CTRCD was mild in 32 and moderate in 10 patients, defined as a change in cardiac biomarkers or GLS and LVEF decline < 50% respectively. Overall, median SERPINA3 levels decreased from baseline to three months after AnC (215.7 [62.0–984.0] to 176.9 [94.7–678.0] µg/ml, *p* = 0.031). This decrease was most prominent in patients without CTRCD (30.8% decrease, *p* = 0.007), followed by mild CTRCD (9.0% decrease, *p* = 0.022), while patients with moderate CTRCD did not show a reduction in SERPINA3 (5.1% increase, *p* = 0.987). SERPINA3 values at three months after AnC were positively correlated with NT-proBNP (r = 0.47, *p* = 0.002). Several malignancy, treatment and patient characteristics were associated with higher SERPINA3 values.

**Conclusion:**

Circulating SERPINA3 levels show dynamic changes in a population of patients with cancer, with an overall decrease following AnC. However, in patients that developed moderate CTRCD, SERPINA3 levels remained elevated. The potential of SERPINA3 dynamics as a biomarker for CTRCD, deserves validation in larger cohorts.

**Graphical Abstract:**

Overview of study protocol CTRCD development and SERPINA3 evolution in the study population. Created using Biorender.

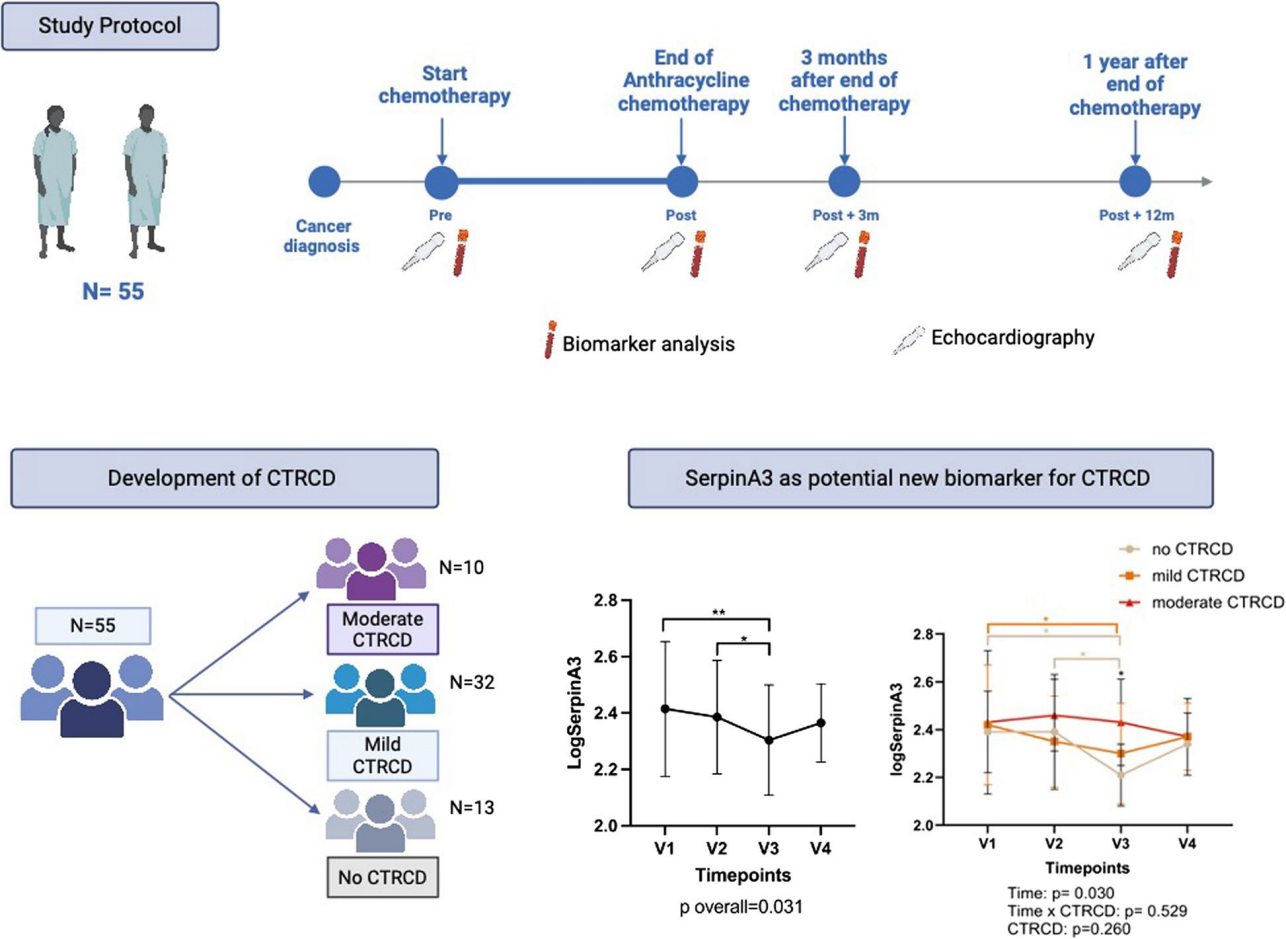

**Supplementary Information:**

The online version contains supplementary material available at 10.1186/s40959-025-00324-7.

## Background

Cancer therapy-related cardiac dysfunction (CTRCD) is an important side effect of anthracycline chemotherapy (AnC). Whereas the original definition of cardiotoxicity was based solely on a decline in left ventricular ejection fraction (LVEF) of > 10% to < 50% [1], the 2022 ESC guidelines on Cardio-Oncology recommend the use of a more broadly encompassing definition for CTRCD [2]. Apart from reduction in LVEF, any new rise in cardiac biomarkers such as cardiac troponin I (cTnI) or T(cTnT) (> 99th percentile), Brain Natriuretic Peptide (BNP) (≥ 35 pg/ml) or N-terminal pro-B-type natriuretic peptide (NT-proBNP) (≥ 125 pg/ml), and/or a relative decline in GLS by ≥ 15% from baseline is now considered as CTRCD [2].

Close monitoring for the development of CTRCD is important to allow for early diagnosis and treatment, but also for guidance of cancer therapy [2, 3]. However, there are some limitations to the use of the classical cardiac biomarkers. Standard cut-offs of these biomarkers are used, which have not been validated in a cancer population. In fact, elevated baseline levels of cTnT and NT-proBNP have been reported in cancer populations, even before the start of chemotherapy [4]. Additionally, these are nonspecific markers of cardiac damage or pressure overload without a specific link to the underlying pathophysiology. Additional biomarkers for CTRCD are therefore needed.

Apart from CTRCD, patients with cancer have an increased lifetime cardiovascular risk and vice versa, heart failure patients are at an increased risk for cancer [5–7] and circulating proteins could modulate this complex interplay [8, 9].

Of particular interest is serpin peptidase inhibitor, clade A member 3 (SERPINA3), also known as alpha-1 antichymotrypsin. Recently, in a murine cardiovascular model, SERPINA3 was found to be upregulated and in a population of cancer survivors with CTRCD (LVEF < 50%), on average 5 years after AnC, SERPINA3 values were increased compared to an age and sex-matched control population [10]. SERPINA3 is expressed in failing hearts and has been described as a prognostic marker for both de novo and worsening heart failure with reduced ejection fraction [8, 11–13]. Elevated plasma levels are associated with increased all-cause mortality in heart failure [11]. Increased myocardial expression of the *SERPINA3* gene has been described as a source of increased circulating SERPINA3 in cardiac conditions [11, 14].

SERPINA3 is part of a large family of irreversible serine protease inhibitors, known as serpins. Overall, SERPINA3 is regarded as an acute phase protein, but its exact function is not yet fully understood. SERPINA3s has anti-inflammatory properties by targeting neutrophilic cathepsin G [15], a component of neutrophilic granules, released during inflammation [16]. Contrary to this, SERPINA3 can directly stimulate release of interleukins by endothelial cells, having pro-inflammatory effects. Next to its protease-inhibiting properties SERPINA3 is the only serpin able to bind to DNA [17]. The binding of SERPINA3 to DNA leads to chromatin condensation, inhibition of DNA polymerase and finally a decrease in DNA synthesis. This can result in an inhibition of cellular proliferation, growth, and differentiation [18]. In myocardial tissue, SERPINA3 was linked to NF- κB activation, resulting in inflammation, oxidative stress and apoptosis [19].

Additionally, SERPINA3 has known proliferative properties [8] which is important in the setting of a cancer diagnosis. In leukaemia and lymphoma patients, circulating SERPINA3 was described to be 1.2-fold elevated compared to controls [20, 21]. In breast cancer tissue, SERPINA3 was also found upregulated, and it seems to promote cell proliferation, migration, and invasion [22].

In the current work we aimed to prospectively investigate the dynamics of circulating SERPINA3 in a cancer population treated with AnC and its relation with CTRCD.

## Methods

### Patient cohort

In this single centre cohort study, adult patients with cancer requiring AnC were enrolled between 01/2020 and 12/2022. Patients had to be > 18-year-old at start of treatment and had to receive AnC. Both breast cancer and haematological malignancies (leukaemia or lymphoma) were included. If patients missed at least two follow-up visits, they were excluded from the analysis. Patients with previous CTRCD or reduced LVEF at baseline were excluded. Previous cancer and cancer therapy did not serve as an exclusion criterion, but if AnC was given, this was accounted for in total doxorubicin equivalent dose. Total AnC dose was calculated as doxorubicin equivalent dose [23]. Treatment-related (type and dose of AnC and concomitant treatment) and clinical risk factors were assessed at baseline.

Analysis for cardiac function and biomarkers took place at 4 timepoints: at baseline (V1), at the end of AnC (V2), three months after the end of AnC (V3) and 1 year after the end of AnC (V4) (Graphical abstract).

The study complies with the Declaration of Helsinki, was approved by the local ethics committee, and all subjects gave written informed consent.

### SERPINA3 levels and other biomarkers

SERPINA3 levels were analysed in batch on stored plasma samples using a commercially available ELISA kit (E-80CYT, Immunology Consultants Laboratory, Inc.), according to the manufacturer’s instructions. Cardiac troponin I (hs-cTnI) and NT-proBNP were measured in serum at the day of sampling.

### Cardiac function

A comprehensive transthoracic echocardiographic evaluation of systolic and diastolic function, ventricular and atrial geometry, was performed on a Vivid E9 cardiovascular ultrasound machine (GE Healthcare, Norway). Systolic left ventricular function was assessed using 3D LVEF measurement and global longitudinal strain (GLS). Echocardiography was performed by experienced sonographers and data were analysed offline by one single experienced sonographer blinded to the study visits using dedicated software (EchoPAC, GE Medical Systems, Norway) [24].

CTRCD was defined according to ESC guidelines [2]. Mild CTRCD was defined as a rise in biomarkers from baseline (hs-cTnI > 45 ng/l and/or NT-proBNP > 125 pg/ml) and/or a rise in GLS with more than 15% from baseline. Moderate CTRCD was defined as a decline in LVEF to 40–49%.

### Statistical analysis

Normality testing was assessed using Shapiro–Wilk test and visual inspection of QQ-plots. Normally distributed data are presented as mean ± standard deviation and skewed data is presented as median [range].

One-way Anova was used for comparisons of continuous variables with normal distribution between CTRCD groups. Kruskal–Wallis test was used for comparisons of skewed continuous variables. Chi-square test was used for comparisons of categorical variables. Correlations were assessed using Pearson coefficients for two normally distributed variables and Spearman coefficients for skewed continuous variables.

Variation over time of continuous variables was assessed with linear mixed models with Timepoint and CTRCD-group as fixed factors and random intercept per subject. Model diagnostics were assessed using residual plots. For skewed data, log-transformation was applied if appropriate. Categorical variables were used as factors, if interaction terms were not significant, they were excluded from the model. For biomarkers, the logarithmically transformed data are displayed in the figures. When significant effects were found, post-hoc comparisons were made to study where differences were situated. Given the small sample size and exploratory nature of the trial, no correction for multiple testing was applied. Discriminatory properties of biomarkers for identification of moderate CTRCD were assessed using ROC curves.

All analyses were performed using SPSS Statistics version 28 (IBM Corporation). A two-sided *p-*value of < 0.05 was considered statistically significant. Graphs were made using GraphPad Prism version 10.1.1.

## Results

### Baseline characteristics and prevalence of CTRCD

A total of 55 patients fulfilled inclusion criteria. The majority of patients was female (45, 81.8%). Most patients presented with breast cancer (70.9%), a minority had leukaemia (18.2%) or lymphoma (10.9%). The most frequently used AnC was doxorubicin followed by daunorubicin (respectively 42 and 10 patients), 2 patients received epirubicin and 1 patient received mitoxantrone. For breast cancer patients the median cumulative dose was 240 mg/m^2^ [120–240 mg/m^2^], for leukaemia 180 mg/m^2^ [72–259 mg/m^2^] and for lymphoma 300 mg/m^2^ [160–300 mg/m^2^]. Duration of AnC was 8 weeks for breast cancer patients (4 cycles every two weeks), 18 weeks for lymphoma (6 cycles every 3 weeks) and three subsequent days during the induction phase for leukaemia. Adjuvant radiotherapy was provided to 39 patients and was directed at the left-sided chest in 21 patients. Additionally, 10 patients received concomitant trastuzumab (18.2%) and 3 received pertuzumab (5.5%).

Of the included patients, 1 patient missed the visit at V2, 6 patients missed the visit at V3 and for 17 patients no data at V4 were available.

**Fig. 1:**
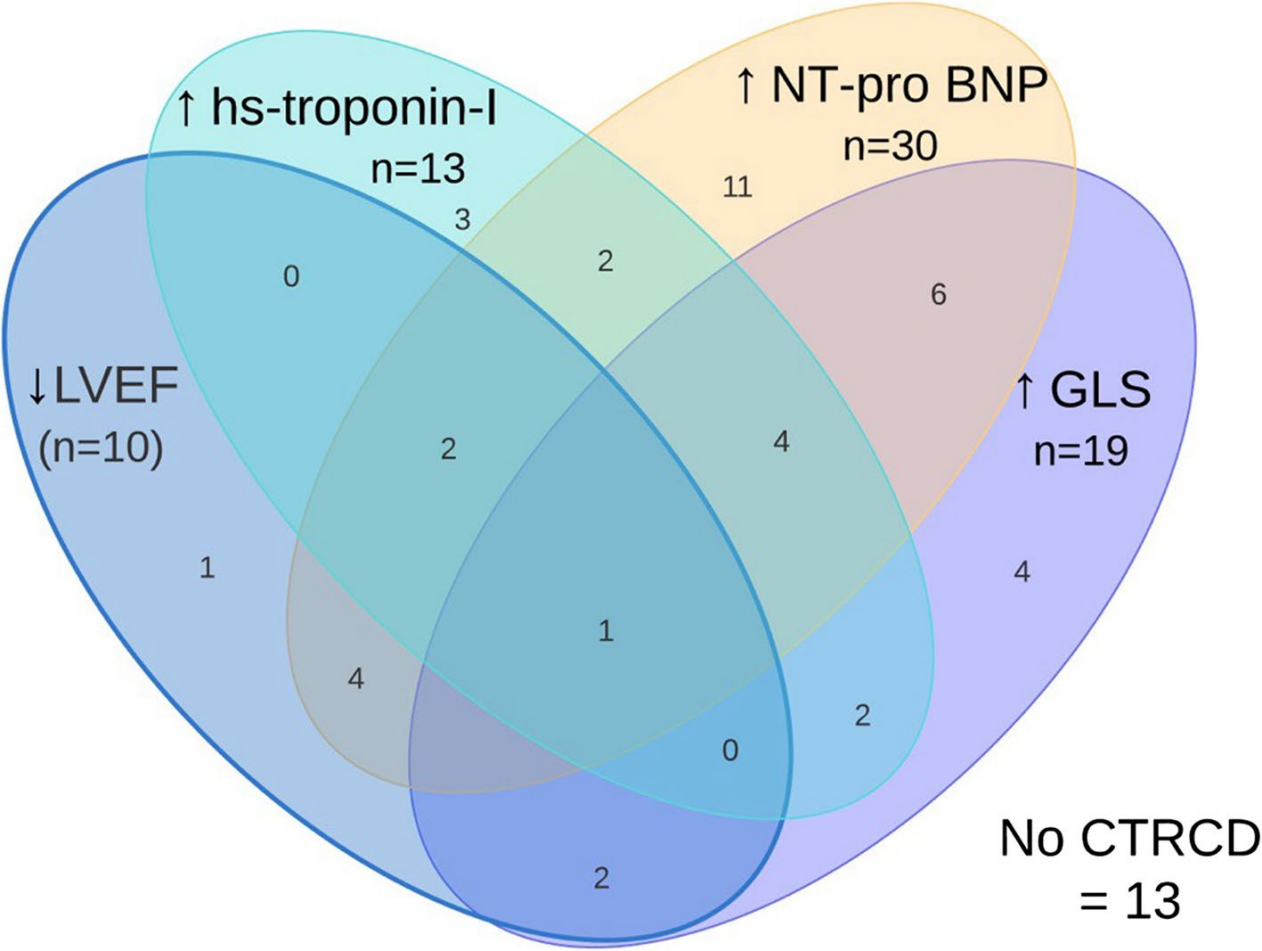
Overview of patients with criteria for the diagnosis of CTRCD. Moderate CTRCD was defined as a decline in LVEF to 40–49%. Mild CTRCD was defined as a rise in biomarkers from baseline (hs-cTnI > 45 ng/l and/or NT-proBNP > 125 pg/ml) and/or a rise in GLS with more than 15% from baseline. CTRCD: Cancer therapy related cardiac dysfunction, LVEF: left ventricular ejection fraction, hs-cTnI: highly sensitive cardiac troponin I; GLS: global longitudinal strain; NT-proBNP: NT-pro brain natriuretic peptide. Created with Lucidchart

Of the 55 included patients, 42 (76.4%) developed asymptomatic CTRCD during follow-up. The first sign of CTRCD was seen at V2 in 33 patients, at V3 in 8 patients and at V4 in one patient. The majority, 32 patients (58.2%), had mild CTRCD, while 10 patients (18.2%) developed moderate CTRCD (Fig. 1). At baseline, LVEF was lower in patients who developed moderate CTRCD (55.6% ± 3.6) compared to patients without CTRCD (60.0% ± 8.1; *p* = 0.03) and patients with mild CTRCD (61.3% ± 5.5; *p* = 0.016), but GLS was normal (≤ −16) in all 3 groups at baseline. No patients developed symptomatic heart failure. No differences were observed in baseline characteristics between groups (Table 1). There were no differences between CTRCD groups in malignancy type (*p* = 0.420), AnC used (*p* = 0.257), total AnC dose (*p* = 0.698), the use of concomitant left-sided radiotherapy (*p* = 0.400) or trastuzumab (*p* = 0.127) (Table 1). Patients who developed moderate CTRCD were treated for either breast cancer (*n* = 7) or leukaemia (*n* = 3).

**Table 1:** Baseline characteristics of the patient cohort according to CTRCD group

	**Overall (*n* = 55)**	**No CTRCD (*n* = 13)**	**Mild CTRCD (*n* = 32)**	**Moderate CTRCD (*n* = 10)**	***p-*value (different CTRCD groups)**
**Demographics**					
**Current age (y)**	53.2 ± 11.5	48.5 ± 7.5	52.0 ± 12.6	47.3 ± 16.6	*P* = 0.247
**Female**	45 (81.8%)	12 (92.3%)	25 (78.1%)	8 (80%)	*P* = 0.528
**BMI (kg/m**^**2**^**)**	25.1 ± 3.3	25.5 ± 2.7	24.8 ± 3.7	25.5 ± 2.6	*P* = 0.706
**Systolic BP (mmHg)**	131.4 ± 17.3	128.6 ± 14.4	135.8 ± 18.0	121.2 ± 14.4	***P* = 0.049**
**Diastolic BP (mmHg)**	80.0 ± 11.4	80.1 ± 11.2	80.5 ± 12.0	77.9 ± 10.8	*P* = 0.821
**Echocardiography**					
**LVEF (%)**	60.3 ± 6.1	60.0 ± 8.1	61.3 ± 5.5	55.6 ± 3.6 *	***P* = 0.041**
**GLS (%)** ^**§**^	−19.4 ± 1.9	−18.4 ± 1.7	−19.6 ± 2.0	−19.2 ± 1.3	*P* = 0.397
**Biochemic analysis**					
**Hs-cTnI (ng/l)**	< 3 [< 3–34]	< 3 [< 3–8]	< 3 [< 3–16]	< 3 [2–8]	*P* = 0.809
**NT-proBNP (pg/ml)**	103[< 35–1335]	86 [34–1335]	104 [34–514]	85 [34–457]	*P* = 0.589
**CRP (mg/l)**	< 4 [< 4 –290.09]	< 4 [< 4 – 93.0]	< 4 [< 4–290.90]	< 4 [< 4–31.0]	*P* = 0.683
**LDL (mg/dl)**	122.9 ± 43.7	112.2 ± 31.2	132.1 ± 50.8	115.7 ± 40.9	*P* = 0.247
**HDL (mg/dl)**	53.0 ± 19.6	55.3 ± 20.6	52.4 ± 20.2	52.1 ± 18.4	*P* = 0.908
**Oncological setting**					
**DOX equivalent dose (mg/m**^**2**^**)**	240 [72–300]	240 [72–240]	240 [120–300]	240 [169–240]	*P* = 0.698
**Radiotherapy**	39 (70.9%)	11 (84.6%)	22 (68.8%)	6 (60%)	*P* = 0.400
**Trastuzumab**	10 (18.2%)	0 (0%)	7 (21.9%)	3 (30%)	*P* = 0.127
**Cardiovascular risk factors**					
**Arterial hypertension**	16 (29.1%)	3 (23.1%)	11 (34.4%)	2 (20%)	*P* = 0.588
**Hypercholesterolemia**	27 (49.1%)	6 (46.2%)	19 (59.4%)	2 (20%)	*P* = 0.091
**Familial history of CVD**	25 (45.5%)	5 (38.5%)	16 (50%)	4 (40%)	*P* = 0.725
**Obesity (BMI > 30 kg/m**^**2**^**)**	4 (7.3%)	0 (0%)	2 (6.3%)	2 (20%)	*P* = 0.189
**Diabetes Mellitus**	3 (5.5%)	0 (0%)	3 (9.4%)	0 (0%)	*P* = 0.320
**Smoker**					*P* = 0.492
**- active**	9 (16.4%)	4 (30.8%)	3 (9.4%)	2 (20%)	
**- previous**	13 (23.6%)	2 (15.4%)	9 (28.1%)	2 (20%)	
**Cardiovascular pharmacotherapy at baseline**					
**Statin**	8 (14.5%)	3 (23.1%)	5 (15.6%)	0 (0%)	*P* = 0.287
**RAAS-inhibitor**	12 (21.8%)	2 (15.4%)	9 (28.1%)	1 (10%)	*P* = 0.391
**Beta-blocker**	7 (12.7%)	2 (15.4%)	5 (15.6%)	0 (0%)	*P* = 0.410

*CTRCD* Cancer therapy related cardiac dysfunction, *BMI* Body Mass Index, *Bpm* beats per minute, *BC* breast cancer, *pt* patients, *DOX* doxorubicin, *EPI* epirubicin, *DAUNO* daunorubicin, *MITO* mitoxantrone, *LVEF* left ventricular ejection fraction, *GLS* global longitudinal strain, *hsTnI* high sensitive troponin I, *NT-proBNP* NT-proBrain natriuretic peptide, *CVD* cardiovascular disease, *HbA1C* haemoglobin A1C, *HDL* high density lipoprotein, *LDL* low density lipoprotein, *RAAS* renin angiotensin aldosterone system

*p<0.05 for moderate CRTRCD compared to mild/no CTRCD; ^§^ GLS values available for 37 patients

Nine patients had previously been treated for malignancies, two patients had received AnC and left sided radiotherapy in the past, and 1 individual had been previously treated with trastuzumab. Cardiovascular pharmacotherapy was initiated when CTRCD developed. Renin–angiotensin–aldosterone system (RAAS) inhibitors were started in 15 patients (27.3%), 7 patients with moderate CTRCD, 7 with mild and 1 without CTRCD due to arterial hypertension. Beta-blockade was started in 6 patients overall (10.1%, 5 mild and 5 moderate CTRCD).

### Evolution of cardiac function during AnC

**Fig. 2:**
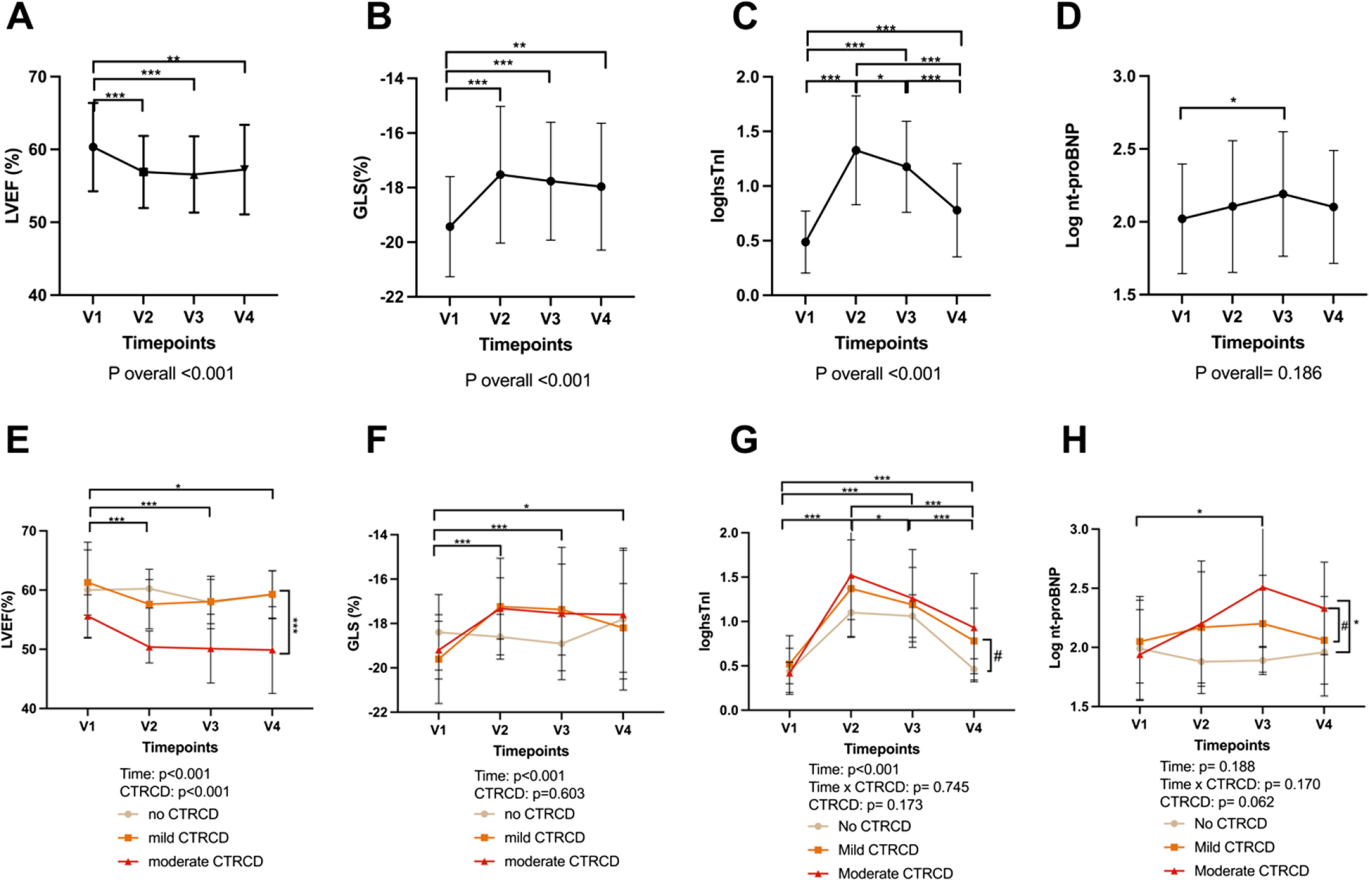
Evolution of echocardiographic and circulating biomarkers during AnC and after chemotherapy. Figure **A**-**D** depict the combined results of the whole study population (*n* = 55). Figure **E**, **F** show the results according to CTRCD group. **A** A significant decline in LVEF over time is seen in up until 12 months after therapy **B** A significant rise in GLS is seen over time. **C** An early peak directly after AnC in hs-cTnI is followed by a decline at 12 months. **D** Three months after AnC, NT-proBNP is significantly higher in patients. **E** A significantly greater decline is present in patients with moderate CTRCD compared to mild and no CTRCD. **F** GLS increases in all CTRCD groups without returning to baseline values. **G** An early peak in hs-cTnI directly after AnC is followed by a subsequent decline at 12 months and is higher in mild and moderate CTRCD. **F** Three months after AnC, NT-proBNP is significantly higher in patients with moderate CTRCD compared to no CTRCD. For hs-cTnI and NT-proBNP statistics was performed on logarithmic transformations. CTRCD: Cancer therapy related cardiac dysfunction, LVEF: left ventricular ejection fraction, hs-cTnI: highly sensitive cardiac troponin I; GLS: global longitudinal strain; NT-proBNP: NT-pro brain natriuretic peptide. #*p* < 0.060, *p < 0.050,**p < 0.010,***p < 0.001

A significant decline in LVEF and a rise in GLS over time was seen in the whole study population up until 12 months after treatment (Fig. 2A-B; E–F).

As shown in Fig. 2E, LVEF was significantly lower at V2 in patients with mild and moderate CTRCD compared to those without CTRCD (no vs moderate: *p* < 0.001; no vs mild *p* = 0.039, mild vs moderate: *p* < 0.001), whereas at V3 and V4, LVEF was significantly lower only in patients with moderate CTRCD compared to those without CTRCD or with mild CTRD (Fig. 2E).

### Dynamic profile of traditional biomarkers during AnC

An early peak of hs-cTnI values directly after AnC was followed by a decline at 12 months (Fig. 2C). This pattern was observed in all CTRCD groups, but values at 12 months post AnC remained increased in mild and moderate CTRCD compared to patients without CTRCD (Fig. 2G).

No overall difference over time in NT-proBNP could be seen, however, at V3 specifically, a significantly higher NT-proBNP level was seen in patients who developed mild and moderate CTRCD compared to those who did not develop CTRCD (no vs moderate: *p* = 0.002; no vs mild *p* = 0.017, mild vs moderate: *p* = 0.148) (Fig. 2 D & H). At twelve months after chemotherapy, values declined, however, without complete normalisation.

### Dynamic profile of SERPINA3 during AnC

**Fig. 3:**
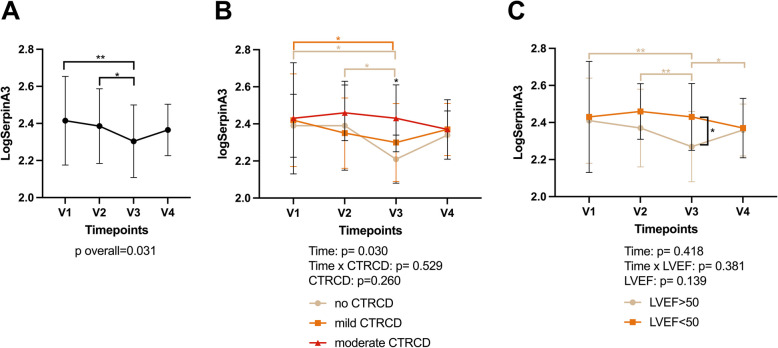
Evolution of SERPINA3 during and after AnC. **A** Evolution of SERPINA3 in the total cohort. SERPINA3 show a dynamic change over time, with return to baseline levels after 12 months. **B** Evolution of SERPINA3 according to CTRCD group. A significant decrease is observed (V1-V3) in patients without and with mild CTRCD, while levels remained unaltered in the moderate CTRCD group. **C** Evolution of SERPINA3 according to presence of a clinically significant decrease in LVEF during follow-up. Only patients with preserved LVEF showed a clinically significant decrease in SERPINA3. CTRCD: cancer therapy related cardiac dysfunction, LVEF: left ventricular ejection fraction. *P < 0.05, **p < 0.01

Overall, SERPINA3 values were significantly decreased 3 months after AnC (V3) compared to baseline (*p* = 0.004) (Fig. 3A).

When patients were divided into CTRCD groups, a significant decline from V1 to V3 was observed in patients with no or mild CTRCD, whereas SERPINA3 levels remained elevated at V3 in patients that had developed moderate CTRCD. At V3, absolute values of SERPINA3 were higher in moderate CTRCD (248 [158–533]μg/ml, *p* = 0.009) compared to no CTRCD (157 [95–264] μg/ml) or mild CTRCD (165 [110–678]μg/ml) (Fig. 3B).

Since the evolution of SERPINA3 values was very similar between patients without CTRCD or with mild CTRCD, we then repeated the analysis by dividing the patients into patients with a clinically significant decline in LVEF to < 50%, warranting treatment, (*N* = 10; moderate CTRCD) and patients without a clinically significant decline in LVEF (LVEF ≥ 50% at all times during follow-up = mild and no CTRCD). Again, this showed a significant decline in SERPINA3 values in patients without a clinically significant decline in LVEF to < 50%, whereas the evolution of SERPINA3 values over time was opposite in patients with a decline in LVEF to < 50% (Fig. 3C). Overall, one year after AnC (V4) SERPINA3 values no longer differed between CTRCD groups.

Of the 10 patients with moderate CTRCD, five showed complete recovery during follow-up (defined as normalisation of LVEF, GLS and biomarkers). There were no significant differences in SERPINA3 values in patients with or without recovery.

**Fig. 4:**
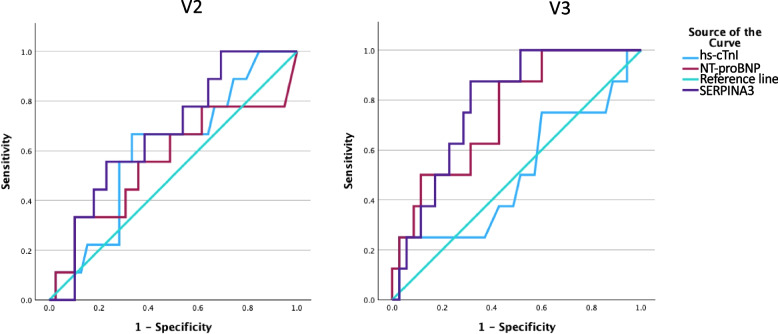
ROC curves for moderate CTRCD (LVEF 40–49%). **A** SERPINA3 has the highest AUC at V2 compared to NT-proBNP and hs-cTnI. **B** SERPINA3 has the highest AUC at V3 compared to NT-proBNP and hs-cTnI. CTRCD: Cancer therapy related cardiac dysfunction, LVEF: left ventricular ejection fraction, hs-cTnI: highly sensitive cardiac troponin I; NT-proBNP: NT-pro brain natriuretic peptide

In a mixed model for SERPINA3 according to time and CTRCD group, CTRCD could not be withheld as a significant factor, possible due to insufficient power in this small, hypothesis-generating cohort. Interestingly, NT-proBNP and hs-cTnI, generally accepted as biomarkers for CTRCD, and included in the definition of mild CTRCD [2], also did not show overall significant differences between patients with no, mild and moderate CTRCD when assessed in a mixed model (Supplemental Table 1). SERPINA3 showed greatest discriminating value between moderate CTRCD versus mild/no CTRCD at V2 (AUC of ROC curve 0.670; *p* = 0.064) compared to hs-cTnI (AUC 0.605; *p* = 0.296) and NT-proBNP (AUC 0.561; *p* = 0.610) and at V3(AUC 0.786 (*p* < 0.001) vs 0.507 (*p* = 0.953) and 0.750 (*p* = 0.004) respectively) (Fig. 4 A, B).

### Association of SERPINA3 with cardiac parameters

SERPINA3 was negatively correlated with LVEF (r = −0.35, *p* = 0.016) and positively correlated with NT-proBNP at V3 (r = 0.47, *p* = 0.002). Additionally, SERPINA3 values at V3 were positively correlated with GLS and hs-cTnI values at V2 (r = 0.47, *p* = 0.006 and r = 0.34 *p* = 0.028) (Supplemental Fig. 1).

SERPINA3 positively correlated with C-reactive protein (CRP) at V2, V3 and V4 (r = 0.66, *p* < 0.001; r = 0.56; *p* < 0.001 and r = 0.46, *p* = 0.006 respectively). SERPINA3 values were negatively correlated with lymphocyte count at baseline, V2 and V4 (r = −0.30, *p* = 0.032; r = −0.43, *p* = 0.005 and r = −0.57, *p* = 0.001 respectively) (Supplemental Fig. 2).

### Influence of patient characteristics, cancer type treatment modalities on SERPINA3

**Fig. 5:**
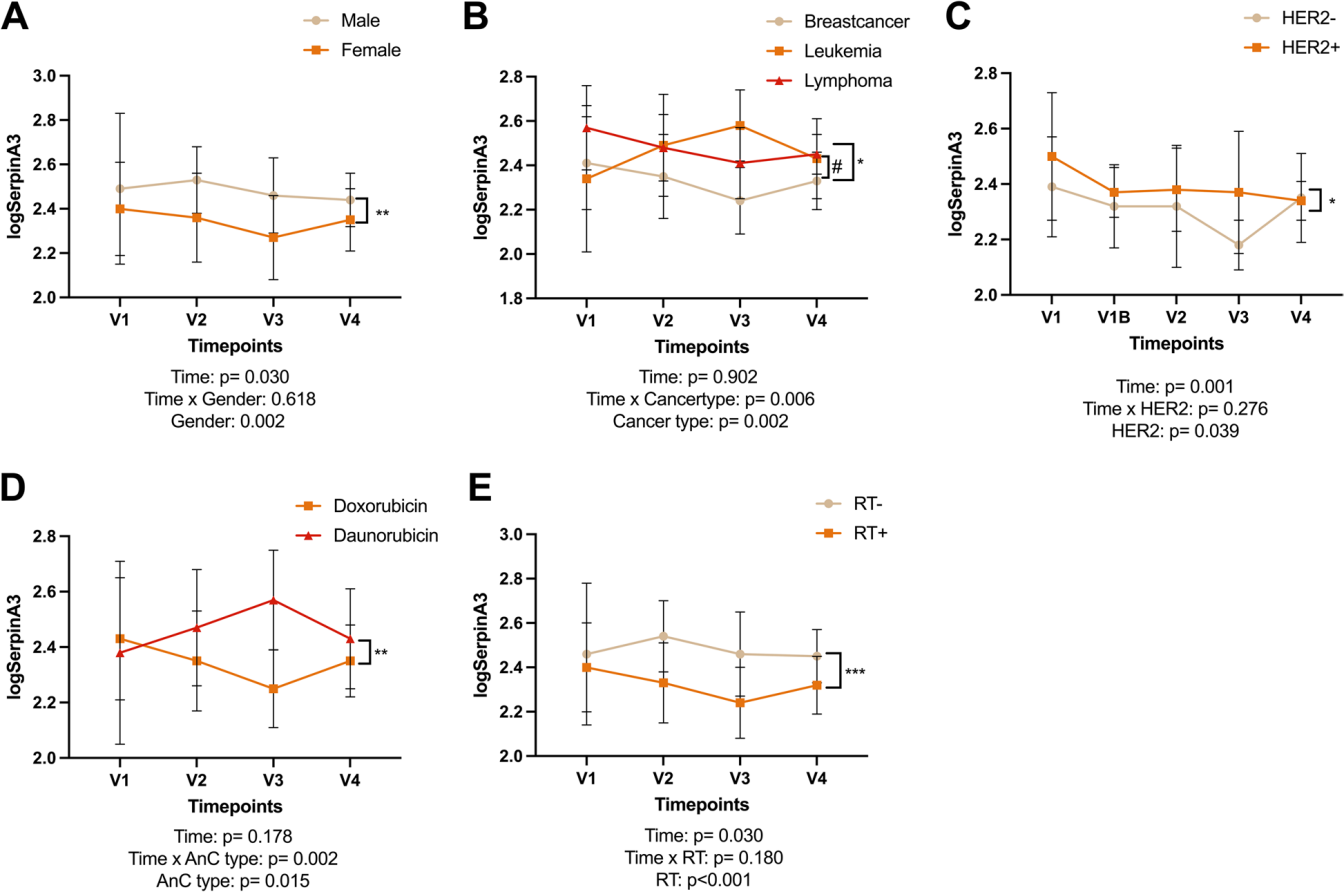
SERPINA3 according to patient characteristics and malignancy. **A** SERPINA3 values are higher in male than females. **B** SERPINA3 values are lower in breast cancer patients compared to leukaemia or lymphoma patients. **C** SERPINA3 values are lower in HER2 negative compared to HER2 positive breast cancer patients. **D** SERPINA3 values in patients treated with doxorubicin declines over time, but increased if patients were treated with Daunorubicin. **E** SERPINA3 values were lower in patients treated with radiotherapy compared to patients who did not receive radiotherapy. HER2: human epidermal growth factor receptor 2; RT: Radiotherapy

There were no significant differences in SERPINA3 values at baseline (V1) between patients with different patient characteristics, such as demographics, cardiac markers including baseline LVEF, cancer type and cardiovascular risk factors (data not shown). However, SERPINA3 values differed over time according to sex, cancer type and treatment modalities. Higher SERPINA3 values were observed in males vs females, haematological cancers vs breast cancer and in HER2 positive vs HER2 negative breast cancer (Fig. 5A, B and C). Regarding treatment modalities, patients who were treated with daunorubicin had overall higher SERPINA3 values than patients treated with doxorubicin and patients who received radiotherapy also had higher values than those who did not (Fig. 5D, E). Lower HDL values were predictive for SERPINA3 values at all timepoints (mixed model; *p* = 0.002).

## Discussion

The present study focuses on assessing the dynamics of circulating SERPINA3 in a population of patients with cancer treated with AnC. Baseline SERPINA3 values were significantly higher in this cancer population (*p* < 0.001) than in our previously reported control cohort [10]. Overall, levels of circulating SERPINA3 declined up to three months after AnC and returned to baseline levels at 12 months after the end of therapy. In contrast, in patients with moderate CTRCD a different evolution in SERPINA3 values was seen, where the decline in SERPINA3 values was absent at three months after chemotherapy.

In our population of 55 cancer patients treated with anthracycline chemotherapy, we showed a high rate of CTRCD (76.4%), defined according to the 2022 ESC guidelines [2]. The majority, 32 patients, showed mild CTRCD, while ten patients (18.2%) developed moderate CTRCD. This is a significantly higher proportion of patients, compared to previously reported cohorts, where an incidence of CTRCD up to 37.5% was observed with 31.6% of patients having mild and up to 9% having moderate or severe CTRCD [25, 26]. The use of GLS and NT-proBNP as parameters for the definition of (mild, often asymptomatic) CTRCD in the current study might partly explain the increased incidence in our study.

Previously, higher SERPINA3 values were observed in a population of cancer survivors with moderate or severe CTRCD [10]. We examined whether SERPINA3 levels are dynamic during AnC and whether these changes are related to CTRCD. Compared to patients with no or mild CTRCD, a different evolution in SERPINA3 values over time was observed in patients with moderate CTRCD. Where SERPINA3 declined significantly after AnC in patients with no or mild CTRCD, an opposite trend with a (non-significant) rise in SERPINA3 levels was observed in patients with moderate CTRCD. Consequently, 3 months after chemotherapy SERPINA3 values were significantly higher in patients with moderate CTRCD compared to patients with no or mild CTRCD. The clinical significance of this trend is not known. We could hypothesize that the decline in SERPINA3 observed is due to a treatment response with tumour decline, in patients with CTRCD this decline might be counteracted by an increase, caused by CTRCD, but this is still purely speculative.

Whereas previous studies that identified SERPINA3 as a prognostic marker in heart failure or ischemic heart disease failed to identify an association between circulating SERPINA3 values and echocardiographic parameters [11, 27], we observed a direct correlation of SERPINA3 values with LVEF and NT-proBNP three months after AnC. SERPINA3 at three months additionally was significantly correlated with hs-cTnI and GLS directly after the end of AnC, which is in line with previous studies in patients with heart failure and CTRCD [10, 28].

We should note that a difference in SERPINA3 dynamics was seen only in patients with moderate CTRCD (defined as a LVEF decline > 10% to < 50%). Whereas for mild CTRCD (based only on cardiac biomarkers and/or GLS) a similar dynamic of SERPINA3 values to patients without CTRCD was seen. Similarly, ROC-analysis showed SERPINA3 to be the best predictor for moderate CTRCD only. Although we observed a high overall incidence of CTRCD, this was only mild in the majority of patients. The clinical relevance of mild CTRCD, which was asymptomatic in all patients, needs to be further determined. In fact, in a population of fourteen patients with moderate to severe CTRCD 5-years after AnC treatment (median LVEF 36% [14–41%]), higher plasma values of SERPINA3 were observed compared to controls. It is plausible that SERPINA3 will only serve as a biomarker for moderate-to-severe CTRCD and differences in SERPINA3 dynamics could be more outspoken in a more severely affected population.

In the present study SERPINA3 was associated with inflammatory markers. Lymphocyte count and CRP were significantly associated with SERPINA3 levels. This is in line with previous reports that described a close association of SERPINA3 with leukocytes and CRP levels [27] and the role of SERPINA3 in inflammation. SERPINA3 expression levels are increased by IL-1, IL-6, and TNF-α [15, 29]. Higher levels of SERPINA3 in failing hearts correlated with higher numbers of infiltrating immune cells [30]. In myocardial samples of patients with left ventricular assist devices, SERPINA3 levels correlated with IL-8 [31]. In the myocardium of patients with dilated cardiomyopathy however, SERPINA3 expression negatively correlated with the number of naïve B cells [32]. Next to being involved in inflammatory response and extracellular matrix remodelling, SERPINA3 has diverse roles and has been described in the regulation of lipid metabolic processes [33]. More specifically, SERPINA3, when combined with amyloid-beta peptide, has been shown to alter intracellular lipid levels, with an increase in uptake and a decrease in degradation of LDL [33]. Whereas LDL and SERPINA3 were not associated in this population, we observed baseline HDL cholesterol value as an independent negative predictor for SERPINA3 level in a mixed model. This confirms a previous report on a negative correlation of HDL with SERPINA3 in a heart failure population [28]. Although total cholesterol was found to be predictive for SERPINA3 values in patients presenting with myocardial infarction previously, we did not observe this in our population [27].

Identifying the driving force behind SERPINA3 dynamics in this population is complex, as SERPINA3 is also linked to cancer and has known proliferative properties [8]. SERPINA3 has been linked to increased tumour aggression, with an increase in tumour migration and invasion in triple negative breast cancer and worse prognosis associated to higher SERPINA3 values [22, 34]. Increased levels of both circulating and tissue-derived SERPINA3 have been described in leukaemia; lymphoma and breast cancer patients, compared to healthy controls [20–22]. However, since a different evolution is seen in patients with moderate CTRCD compared to no or mild CTRCD, we hypothesize that cardiotoxicity is, at least partially, responsible for the change observed. In a preclinical model, AC16 human cardiomyocytes which were treated with doxorubicin showed decreased expression of SERPINA3 [32]. Others have shown that failing hearts show increased expression of SERPINA3 [8, 11, 12], which indicates that SERPINA3 is expressed in myocardial tissue. While initially the dogma was that SERPINA3, being an acute phase protein, is produced by the liver, there are increasing reports suggesting a local cardiac expression of SERPINA3. The exact cellular source remains to be unravelled. Nonetheless, the changes in plasma SERPINA3 observed in our population might at least be partially explained by CTRCD and are likely not solely based on changes in expression in tumour tissue. No cardiac tissue samples of patients in the current study were available to confirm an increased myocardial expression of SERPINA3.However, in a mouse model of DOX-induced cardiovascular toxicity, defined as a decline in LVEF, an increase in arterial stiffness and endothelial dysfunction, an upregulation of SERPINA3 was seen in aortic tissue and cardiac tissue [35, 36]. In myocardium, the upregulation of SERPINA3 was most outspoken in endothelial cells (microvascular) but was also present in cardiomyocytes [35]. Therapy with dexrazoxane prevented the development of cardiovascular toxicity and resulted in absence of the upregulation of SERPINA3 in response to DOX in both tissues [36]. As such a cardiac source of SERPINA3 in the current population seems likely.

A role of SERPINA3 as a prognostic and predictive biomarker for cancer has been previously proposed (reviewed here: [37–39]). It was shown that increased expression of SERPINA3 in tissue from breast cancer patients was associated with stronger proliferation and increased viability of the tumour [22]. SERPINA3 values in tissue correlated with poor prognosis in triple negative breast cancer and were predictive for reduced efficacy of cisplatin chemotherapy [22]. In hormone receptor positive breast cancer on the contrary, increased SERPINA3 values in tissue are associated with a better response to hormone therapy [40]. In patients with acute leukaemia, elevated SERPINA3 in serum correlated with poor overall survival [41]. Additionally, SERPINA3 has also been described as a circulatory biomarker for colorectal cancer and prostate cancer [39]. As such, SERPINA3 might offer the advantage of using a single biomarker able to track both therapy response and development of CTRCD.

Several patient, cancer and treatment characteristics were associated with SERPINA3 values. We observed a significant difference in SERPINA3 values according to sex, cancer type (breast cancer vs haematological malignancies), anthracycline type and the use of radiotherapy (Fig. 4). However, care should be taken when interpreting these results, as the current study was not powered for these merely hypothesis-generating sub-analyses. As all breast cancer patients were female, it is likely that the sex differences are in fact due to the differences in cancer types. This hypothesis is supported by the lack of sex differences in circulating SERPINA3 in different populations [11].

SERPINA3 values at the 12-month post-treatment endpoint were similar across groups and seemed to return to baseline values. We do not have a binding explanation for this evolution. The smaller sample size at this timepoint could certainly have affected this observation, due to mortality and dro*p-*out, at this time point could certainly have affected this observation. Additionally, Six patients in the cohort had disease progression at this point, which could have resulted in a new increase in SERPINA3 values.

### Future perspectives

A larger prospective follow-up study is needed to confirm the dynamics of SERPINA3 and its relationship with CTRCD after anthracycline chemotherapy. Attention should be given to standardization of cancer subtype and treatment (DOX dose equivalent, concomitant treatment) to decrease the influence of cofounding factors on the relationship between SERPINA3 values and CTRCD. Moreover, it would be of interest to investigate if SERPINA3 values diverge between CTRCD groups early on in treatment, between chemotherapy cycles. Finally, assessment of normal values of circulating SERPINA3 and its variation in healthy controls and cancer patients are needed to provide clinically useful cut-off values.

## Conclusions

In a cancer population, circulating SERPINA3 levels show a dynamic response following AnC. In the overall study population, a decrease in SERPINA3 is apparent within 3 months after the end of AnC, possibly reflecting normalisation of levels due to cancer treatment. However, in patients who develop moderate CTRCD, SERPINA3 values remain unchanged. SERPINA3 values are linked to several patient, cancer and treatment related characteristics and a clear association between SERPINA3 values and inflammatory markers is present, justifying further investigation of its role as a diagnostic and prognostic biomarker in CTRCD.

## Supplementary Information

Supplementary Material 1: Supplemental Table 1. Regression tables of mixed model analysis with covariates p1. Supplemental Fig. 1. Correlation of SERPINA3 with Cardiac parameters p6. Supplemental Fig. 2. Correlation of SERPINA3 with inflammatory parameters p8

## Data Availability

The datasets used and/or analysed during the current study are available from the corresponding author on reasonable request.

## Abbreviations

AnC: Anthracycline chemotherapy
BMI: Body mass index
NT-proBNP: N-terminal pro-B-type natriuretic peptide
CRP: C-reactive protein
hs-cTnI: High-sensitive cardiac troponin I
hs-cTnT: High-sensitive cardiac troponin T
CTRCD: Cancer therapy-related Cardiac Dysfunction
DBP: Diastolic blood pressure
GLS: Global longitudinal strain
HbA1C: Haemoglobin A1c
HDL: High density lipoprotein
HER2: Herceptin receptor 2
HR: Heart rate
LVEF: Left ventricular ejection fraction
MBP: Mean blood pressure
MRA: Mineralocorticoid receptor antagonist
SBP: Systolic blood pressure
SERPINA3: Serpin peptidase inhibitor, clade A member 3
WBC: White blood cell count
