# Supplementary material for: Dynamics of SERPINA3 in response to anthracycline treatment and cardiovascular dysfunction
Source: Cardiooncology. 2025 Mar 14;11:27. doi: 10.1186/s40959-025-00324-7 (PMC11907982; doi:10.1186/s40959-025-00324-7)
Supplement: Supplementary file 1 — Supplementary Material 1: Supplemental Table 1. Regression tables of mixed model analysis with covariates p1. Supplemental Fig. 1. Correlation of SERPINA3 with Cardiac parameters p6. Supplemental Fig. 2. Correlation of SERPINA3 with inflammatory parameters p8 [file 40959_2025_324_MOESM1_ESM.pdf]

Supplementary data

1. Supplemental Table 1: Regression tables of mixed model analysis with covariates p1.
2. Supplemental Figure 1: Correlation of SERPINA3 with Cardiac parameters p6.
3. Supplemental Figure 1: Correlation of SERPINA3 with inflammatory parameters p8.

Supplemental Table 1: Regression tables of mixed model analysis with covariates.

**Predictor variable:**	**Coefficient/ mean**	**Standard error**	**t-value**	**p-value**	**Confidence interval**	**AIC**
**LVEF by Timepoint**						1185.527
Intercept	57.383	0.879	65.302	**P<0.001**	55.648 – 59.118	
Timepoint				**P<0.001**		
V1	60.327	0.754			58.835 – 61.820	
V2	56.927	0.760			55.425 – 58.429	
V3	56.632	0.788			55.074 – 58.190	
V4	57.383	0.879			55.648 – 59.118	
**GLS by Timepoint**						**614.857**
Intercept	-17.833	0.424	-42.046	**P<0.001**	-18.672 - -16.995	
Timepoint				**P<0.001**		
V1	-19.413	0.358			-20.121 - -18.705	
V2	-17.402	0.354			-18.103 - -16.702	
V3	-17.738	0.339			-18.410 - -17.066	
V4	-17.833	0.424			-18.672 - -16.995	
**Log hs-cTnI by Timepoint**						**165.038**
Intercept	0.772	0.064	12.034	**P<0.001**	0.645 – 0.899	
Timepoint				**P<0.001**		
V1	0.508	0.57			0.395 – 0.621	
V2	1.325	0.056			1.213 – 1.437	
V3	1.178	0.059			1.061 – 1.294	
V4	0.772	0.064			0.645 – 0.899	
**Log NT-proBNP by Timepoint**						**160.524**
Intercept	2.081	0.065	32.099	**P<0.001**	1.953 – 2.209	
Timepoint				P=0.186		
V1	2.047	0.059			1.931 – 2.164	
V2	2.110	0.058			1.995 – 2.225	
V3	2.178	0.060			2.058 – 2.297	
V4	2.081	0.065			1.953 – 2.209	
**LVEF by Timepoint with CTRCD**						**1144.502**
Intercept	51.320	1.145	44.831	**P<0.001**	49.045 – 53.594	
Timepoint				**P<0.001**		
V1	59.289	0.676			57.954 – 60.623	
V2	55.893	0.680			54.550 – 57.235	
V3	55.583	0.712			54.178– 56.988	
V4	56.492	0.811			54.892 – 58.093	
CTRCD				**P<0.001**		
No	59.796	0.878			58.038 – 61.555	
Mild	59.004	0.536			57.926 – 60.082	
Moderate	51.642	0.953			49.724 – 53.559	
Timepoint x CTRCD				P=0.217		
**GLS by Timepoint with CTRCD**						**612.071**
Intercept	-17.574	0.6660	-26.629	**P<0.001**	-18.894 - -16.254	
Timepoint				**P<0.001**		
V1	-19.448	0.400			-20.240 - -18.656	

V2	-17.432	0.392			-18.209 - -16.656
V3	-17.774	0.374			-18.516 - -17.032
V4	-17.892	0.452			-18.786 - -16.998
CTRCD				P=0.663	
Timepoint x CTRCD				P=0.366	
**Log hs-cTnI by Timepoint with CTRCD**					**166.560**
Intercept	0.868	0.112	7.774	P<0.001	0.645 – 1.090
Timepoint				**P<0.001**	
V1	0.504	0.061			0.383 – 0.626
V2	1.322	0.061			1.202 – 1.442
V3	1.171	0.063			1.047 – 1.296
V4	0.760	0.068			0.625 – 0.895
CTRCD				P=0.173	
Timepoint x CTRCD				P=0.745	
**Log NT-proBNP by Timepoint with CTRCD**					**159.777**
Intercept	2.211	0.114	19.403	**P<0.001**	1.983 – 2.438
Timepoint				P=0.188	
CTRCD				P=0.062	
Timepoint x CTRCD				P=0.170	
**logSERPINA3 by timepoint**					**-50.753**
Intercept	2.372	0.034	69.968	**P<0.001**	2.305 – 2.439
Timepoint				**P=0.031**	
V1	2.415	0.027			2.361 – 2.469
V2	2.386	0.029			2.330 – 2.443
V3	2.305	0.030			2.246 – 2.364
V4	2.372	0.034			2.305 – 2.439
**logSERPINA3 by timepoint with CTRCD**					**-44.676**
Intercept	2.424	0.049	49.758	**P<0.001**	2.327 – 2.521
Timepoint				**P=0.030**	
V1	2.421	0.029			2.365 – 2.478
V2	2.392	0.030			2.334 – 2.451
V3	2.311	0.031			2.250 – 2.372
V4	2.374	0.035			2.305 – 2.444
CTRCD				P=0.260	
Timepoint x CTRCD				P=0.529	
**logSERPINA3 by timepoint with LVEF<50%**					**-48.711**
Intercept	2.425	0.048	50.112	**P<0.001**	2.329 – 2.521
Timepoint				P=0.418	
LVEF<50%				P=0.139	
Timepoint x LVEF<50%				P=0.381	
**logSERPINA3 by timepoint with Sex**					**-55.843**
Intercept	2.343	0.034	68.815	**P<0.001**	2.276 – 2.411
Timepoint				**P=0.030**	

V1	2.458	0.030			2.400 – 2.517	
V2	2.432	0.031			2.370 – 2.494	
V3	2.349	0.032			2.286 – 2.413	
V4	2.412	0.036			2.342 – 2.482	
Sex				**P=0.002**		
Male	2.481	0.039			2.403 – 2.559	
Female	2.344	0.018			2.308 – 2.381	
Timepoint x Sex				**P=0.618**		
**logSERPINA3 by timepoint with Cancertype**						**-54.814**
Intercept	2.453	0.085	28.912	P<0.001	2.285 – 2.620	
Timepoint				P=0.902		
Cancertype				P=0.002		
Breast cancer	2.333	0.019			2.294 – 2.372	
Leukaemia	2.465	0.040			2.384 – 2.546	
Lymphoma	2.479	0.048			2.381 – 2.576	
Timepoint x Cancertype				P=0.006		
V1 x Breast cancer	2.411	0.030			22.351 – 2.471	
V1 x Leukaemia	2.335	0.060			2.217 – 2.454	
V1 x Lymphoma	2.573	0.078			2.420 – 2.726	
V2 x Breast cancer	2.348	0.032			2.286 – 2.411	
V2 x Leukaemia	2.487	0.063			2.362 – 2.612	
V2 x Lymphoma	2.478	0.085			2.311 – 2.646	
V3 x Breast cancer	2.236	0.033			2.172 – 2.301	
V3 x Leukaemia	2.591	0.077			2.439 – 2.743	
V3 x Lymphoma	2.411	0.078			2.258 – 2.564	
V4 x Breast cancer	2.337	0.038			2.261 – 2.413	
V4 x Leukaemia	2.447	0.077			2.295 – 2.599	
V4 x Lymphoma	2.453	0.085			2.285 – 2.620	
**logSERPINA3 by timepoint with HER2**						**-80.906**
Intercept	2.409	0.045	54.015	**P<0.001**	2.320 – 2.497	
Timepoint				**P=0.001**		
V1	2.435	0.030			2.375 – 2.496	
V2	2.356	0.031			2.294 – 2.412	
V3	2.256	0.033			2.191 – 2.321	
V4	2.368	0.037			2.294 – 2.442	
HER2				**P=0.039**		
Yes	2.394	0.031			2.330 – 2.458	
No	2.313	0.021			2.270 – 2.356	
Timepoint x HER2				P=0.276		
**logSERPINA3 by timepoint with AnC Type**						**-58.926**
Intercept	2.314	0.191	12.129	**P<0.001**	1.937 – 2.690	
Timepoint				P=0.178		
AnC type				**P=0.015**		

Doxorubicin	2.345	0.019			2.306 – 2.383	
Daunorubicin	2.469	0.041			2.387 – 2.552	
Timepoint x AnC type				**P=0.002**		
V1 x Doxorubicin	2.426	0.029			2.368 – 2.484	
V1 x Daunorubicin	2.381	0.060			2.262 – 2.500	
V2 x Doxorubicin	2.350	0.031			2.289 – 2.411	
V2 x Daunorubicin	2.469	0.063			2.344 – 2.595	
V3 x Doxorubicin	2.248	0.031			2.187 – 2.309	
V3 x Daunorubicin	2.578	0.077			2.426 – 2.730	
V4 x Doxorubicin	2.355	0.036			2.284 – 2.425	
V4 x Daunorubicin	2.449	0.077			2.297 – 2.601	
**logSERPINA3 by timepoint with RT**						**-64.094**
Intercept	2.316	0.034	68.148	**P<0.001**	2.249 – 2.383	
Timepoint				**P=0.030**		
V1	2.447	0.027			2.394 – 2.499	
V2	2.419	0.028			2.364 – 2.474	
V3	2.337	0.029			2.280 – 2.394	
V4	2.392	0.033			2.328 – 2.457	
RT				**P<0.001**		
Yes	2.323	0.018			2.287 – 2.359	
No	2.475	0.027			2.420 – 2.530	
Timepoint x RT				0.180		
**logSERPINA3 by timepoint with HDL**						**-58.550**
Intercept	2.508	0.055	45.360	**p<0.001**	2.398 - 2.618	
Timepoint				**P=0.025**		
V1	2.410	0.027			2.357 – 2.463	
V2	2.380	0.028			2.325 – 2.435	
V3	2.299	0.028			2.243 – 2.355	
V4	2.363	0.033			2.298 – 2.427	
HDL	-0.0027	0.0008	-3.218	**0.002**	-0.0044 - -0.0010	
Timepoint x HDL				**0.360**		

If interaction factors were non-significant, mixed models without the interaction factor are displayed.

Supplemental Figure 1

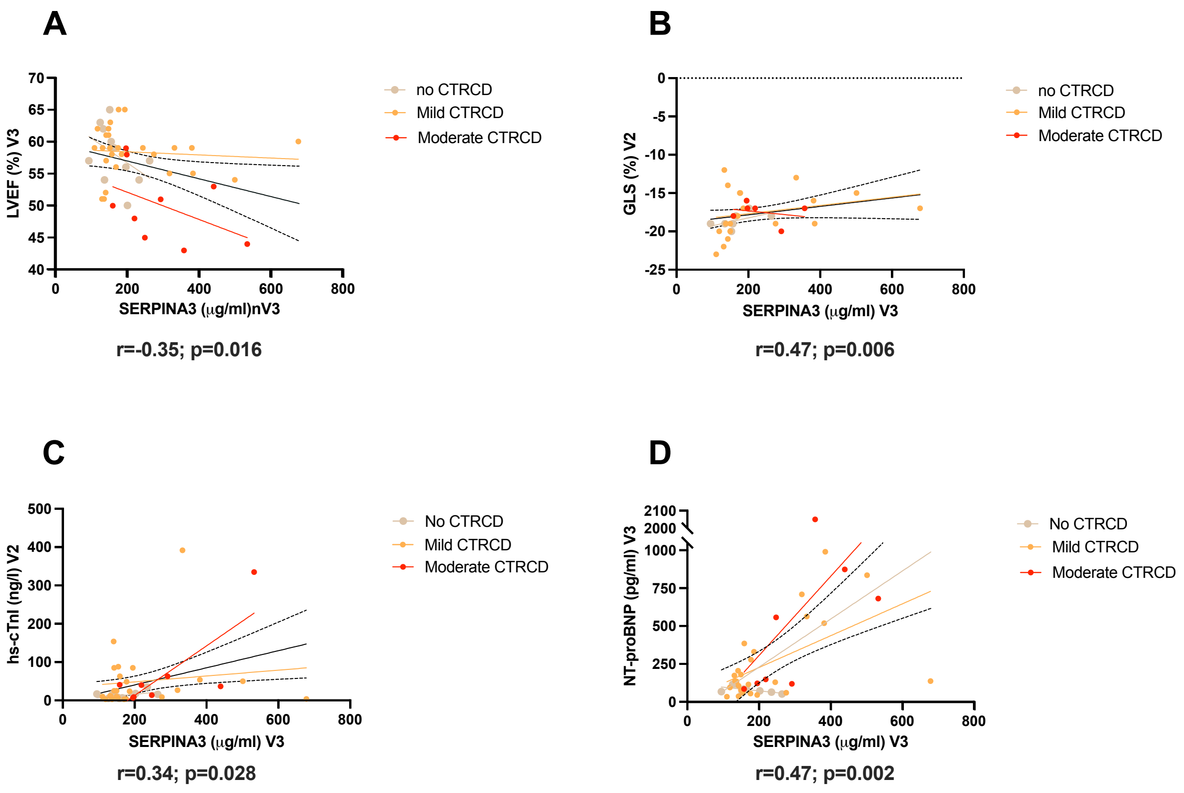

Supplemental Figure 1 Correlations between SERPINA3 at three months post chemotherapy and cardiac markers. *A global correlation of all patients (n=55) is displayed. Individual values are displayed as brown (no CTRCD), yellow (mild CTRCD) or red (moderate CTRCD).* ***A.*** *A significant negative correlation between LVEF and SERPINA3 values at three months after the end of AnC is seen.* ***B****. A significant positive correlation between GLS, directly after the end of treatment, and SERPINA3 values at three months after the end of AnC is seen.* ***C****. A significant correlation between hs-cTnI directly after the end of AnC and SERPINA3 values at three months after the end of AnC is seen.* ***D.*** *A significant correlation between NT-proBNP and SERPINA3 values at three months after the end of AnC is seen. AnC: Anthracycline chemotherapy; CTRCD: Cancer therapy related cardiac dysfunction, LVEF: left ventricular*

ejection fraction, hs-cTnI: highly sensitive cardiac troponin I; GLS: global longitudinal strain; NT-proBNP: NT-pro brain natriuretic peptide.

Supplemental Figure 2

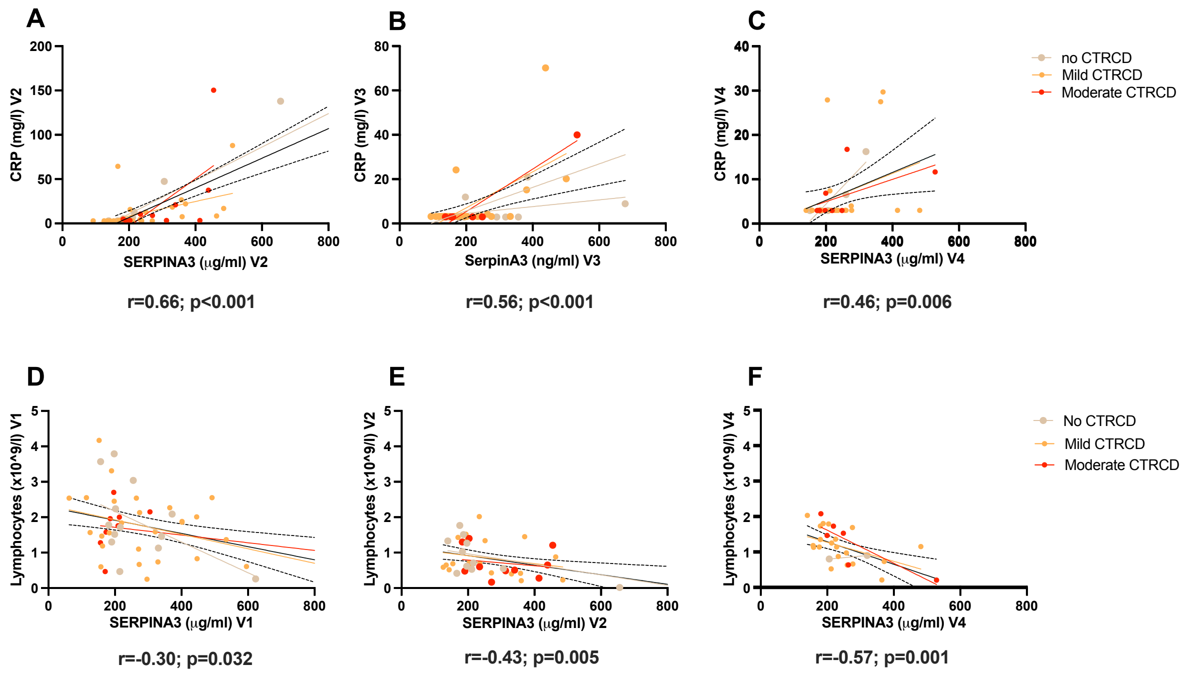

Supplemental Figure 2 Correlations between SERPINA3 and inflammatory markers at different tiemoints. *A global correlation of all patients (n=55) is displayed. Individual values are displayed as brown (no CTRCD), yellow (mild CTRCD) or red (moderate CTRCD).* ***A-C****: Correlation between CRP and SERPINA3 at different timepoints.* ***A.*** *A significant correlation between CRP and SERPINA3 values directly after the end of AnC is seen.* ***B****. A significant correlation between CRP and SERPINA3 values three months after the end of AnC is seen* ***C****. A significant correlation between CRP and SERPINA3 values one year after the end of AnC is seen.* ***D-F****: Correlation between lymphocyte count and SERPINA3 at different timepoints.* ***D.*** *A significant negative correlation between lymphocyte count and SERPINA3 values at baseline is seen.* ***E.*** *A significant negative correlation between lymphocyte count and SERPINA3 values directly after the end of AnC is seen.* ***F.*** *A significant negative correlation between lymphocyte count and SERPINA3 values one year after the end of AnC is seen. AnC: Anthracycline chemotherapy; CRP: C-reactive protein; CTRCD: Cancer therapy related cardiac*

dysfunction, LVEF: left ventricular ejection fraction, hs-cTnI: highly sensitive cardiac troponin I; GLS: global longitudinal strain; NT-proBNP: NT-pro brain natriuretic peptide.
